# Age-Related Differences in Fixation Pattern on a Companion Robot

**DOI:** 10.3390/s20133807

**Published:** 2020-07-07

**Authors:** Young Hoon Oh, Da Young Ju

**Affiliations:** 1 School of Integrated Technology, Yonsei Institute of Convergence Technology, Yonsei University, 85 Songdogwahak-ro, Yeonsu-gu, Incheon 21983, Korea; 50hoon@yonsei.ac.kr; 2 Department of Future Design, Graduate School of Techno Design, Kookmin University, 77 Jeongneung-ro, Seongbuk-gu, Seoul 02707, Korea

**Keywords:** companion robot, eye tracking, older adult, robot design

## Abstract

Recent studies have addressed the various benefits of companion robots and expanded the research scope to their design. However, the viewpoints of older adults have not been deeply investigated. Therefore, this study aimed to examine the distinctive viewpoints of older adults by comparing them with those of younger adults. Thirty-one older and thirty-one younger adults participated in an eye-tracking experiment to investigate their impressions of a bear-like robot mockup. They also completed interviews and surveys to help us understand their viewpoints on the robot design. The gaze behaviors and the impressions of the two groups were significantly different. Older adults focused significantly more on the robot’s face and paid little attention to the rest of the body. In contrast, the younger adults gazed at more body parts and viewed the robot in more detail than the older adults. Furthermore, the older adults rated physical attractiveness and social likeability of the robot significantly higher than the younger adults. The specific gaze behavior of the younger adults was linked to considerable negative feedback on the robot design. Based on these empirical findings, we recommend that impressions of older adults be considered when designing companion robots.

## 1. Introduction

The rapid increase in the elderly population has led to an increased interest in robotic companions. Many researchers validated the feasibility of a companion robot [1] and highlighted its various benefits. For instance, companion robots provide many health benefits to the elderly by reducing their loneliness [2] and encouraging them to have more social interactions [3].

Many studies not only found benefits of companion robots but also addressed the impacts of the robot’s appearance on older adults [4,5,6]. Although Paro, one of the most representative companion robots, is modeled after an “unfamiliar animal” [7], it was favored by many of its owners. Its soft material, cute face, and body shape were major factors in the strong preference for the robot [7]. Furthermore, Moyle et al. reported that a bear-like robot, CuDDler, was loved by older adults with dementia due to its soft texture and cute appearance [1]. These studies indicate that the design of a companion robot influences older adults’ perceptions of the robot [8,9]. However, several studies indicated [10] that older adults were “considered but not consulted in the development of robots” [11]. Therefore, to design a suitable companion robot, it is critical to understand impressions of older adults [10,12,13].

Previous studies reported that people have different preferences for a robot design depending on their age [14,15,16]. Since most of the studies conducted surveys or interviews to investigate preferences, further objective research is required on which elements of the robot’s appearance are valued by older adults. Thus, this study focused on the age-related differences between younger and older adults. We aimed to capture the physiological responses of the participants using eye-tracking experiments. Moreover, surveys and interviews were conducted to understand the participants’ first impressions of a robot. Our findings revealed that the gazing behavior of the older adults was significantly different from that of the younger adults. The younger adults had more detailed gaze patterns than the older adults and indicated each physical part of the robot in need of improvement. The detailed gaze behavior of the participants seems to be linked with the survey results, and we found that the bear-like robot was preferred significantly more by the older adults.

## 2. Literature Review

### 2.1. Design of a Companion Robot

A huge body of research papers addressed the various benefits of companion robots [2,3,17], and researchers have expanded the scope of research to investigate optimal robot design [18,19,20] for the elderly. For instance, Oh et al. conducted a face-to-face survey of 191 older adults and used stuffed toys to investigate the design preferences of the elderly [16]. Their findings show that the teddy bear-type companion robot was the most preferred design for older adults, except for those aged over 85. Additionally, it was the most preferred design for highly depressed older adults. In another study, which explored the design requirements of a companion robot, Heerink et al. introduced zoomorphic robots to the elderly and observed their responses [21]. The elderly people showed positive responses to the seal-like robot, which might have been due to its soft fur. Indeed, softness has been identified as one of the most important design features of a companion robot [7,10,16,21].

Some researchers used qualitative research methods, mostly based on interviews [6]. Kim et al. interviewed nursing experts and reported that small animal-like robots might be more suitable for elderly than humanoids [22]. Moyle et al. interviewed twenty staff members from long-term care facilities and established the potential of Paro as for older adults with dementia [23]. Through interviews, some researchers observed that robot design was perceived differently depending on the age of the participants. Oh et al. interviewed older and younger adults about five types of companion-robot design [15]. They found a significant age-related difference in robot design preference; older adults preferred animal-like design, while younger adults liked neat and tidy ones.

### 2.2. Eye Tracking for Evaluating the Design of a Product

Eye trackers have been used to investigate a user’s physiological response to a product design [24] because vision plays an important role in the perception of a product [25,26]. In addition, if a user forms a close relationship with a product, its appearance might influence the user experience [4,5,20,27,28,29]. Therefore, it is critical to investigate users’ impressions of the appearance of the product. In this regard, eye trackers have been used to objectively investigate user perceptions of a product design [27].

Most of eye-tracker studies focused on where people are looking at the product [12,26,27,30,31,32,33]. They reported that, when people look at a product, they do not perceive it entirely but only obtain an abstract impression [34]. Their eyes reach salient stimuli first and stay there for a long time [35]. These studies also reported that eye-tracking devices are able to capture the user’s cognitive response and enable the evaluation of the product design [24]. For instance, Ho and Lu showed product images to students and used an eye tracker to investigate the pupil size [26]. When the participants were exposed to negative stimuli, their pupil diameter change was significantly smaller than when they were exposed to positive or neutral stimuli. Based on these results, the authors concluded that pupil size could be used to evaluate product design. Other authors have also proposed that eye-tracking measures could be utilized to evaluate user experience [36] and aesthetics [24,37] of a product. They reported that eye-tracking data such as the number of eye fixations could be used as objective criteria for quantifying or predicting aesthetics. However, limitations of eye tracking have also been reported in the literature [24]. Husić-Mehmedović et al. [30] investigated visual attention paid to beer can packages using eye tracking and a survey. Their findings revealed that the most attractive package from the eye tracker experiment was not necessarily the most likable one in the survey. Eye tracking shows what users see, but its application to the interpretation of the user opinions is limited. In this regard, previous studies concluded that eye-tracking methodology should be complemented by a survey [24,30].

### 2.3. Eye Tracking for Investigating the Design of a Robot

Eye tracking has recently been applied to investigate user perceptions of a robot design. Liu et al. [27] utilized an electroencephalogram, a screen-based eye tracker, and surveys to investigate the preferred designs and attractive features of humanoid robots. They reported that head and face were the prominent features of the robots. Moreover, highly preferred robots tended to draw more attention [27,38,39]. Substantial focus on the robot’s face has also been reported in other studies. Dziergwa et al. examined the gaze behavior of people interacting with a humanoid robot [12]. Among the various physical parts of the robot, participants gazed at the head first and for the longest time. Then, their gaze moved down to the torso and the neck. Similarly, this type of gaze pattern was observed with an animal-like robot. Choi et al. [32] presented the conceptual idea of investigating robot design at a conference by having older and younger adults wear a mobile eye tracker and gaze at a bear-like robot mockup. When the participants looked at the robot for the first time, they focused on the face. This pilot study reported that the elderly looked at the robot’s face significantly more often and a little longer than the younger adults. In their preliminary study, they confirmed that the idea could be a potential research method in robot design, but they could not explain their findings because they did not conduct further research to analyze the differences. They did not analyze gaze plots or eye-tracking recordings either; thus, the interpretation of their findings was strictly limited. Other researchers have also found age-related differences in eye tracking. Park et al. [33] reported that children and younger adults showed significantly different gaze patterns for various robot faces. Based on this result, they concluded that the design of robots should be different depending on the target age group [33]. In summary, several studies adopted the eye tracker to improve the design of robots, but only a few studies have used it to examine age-related differences in perceiving robot design [40] (p. 315), which is one of the contributions of this study.

## 3. Method

### 3.1. Participants

A total of 68 participants volunteered to participate in the study. To investigate age-related differences in looking at the companion robot, we divided them into two groups: older adults aged 50 and older [41,42,43,44] and younger adults aged 30 and younger. However, three older adults (P14, P16, P32) and three young adults (P1, P15, P34) were excluded due to calibration failure that lasted more than 10 min. Therefore, a total of 62 adults, 31 in the older and 31 in the younger group, were included in the analysis (Table 1). Most of the younger adults were undergraduate/graduate students. None of the participants had any history of ocular diseases. All participants had normal or corrected-to-normal visual acuity [33,45,46]. They volunteered to participate in the study and provided informed consent. The study was performed in compliance with the ethical recommendations of the Declaration of Helsinki. They could quit any time during the experiment if they did not wish to continue. All of them were informed that their gaze was to be video-recorded during the eye-tracker experiment [47]. Additionally, interviews that followed the eye-tracking experiment were audio recorded with the consent of all participants and transcribed verbatim. After the end of the study, all participants received about $5 in compensation for their participation.

### 3.2. Companion Robot Mockup

In this study, one inactive bear robot mockup was used as a stimulus for the eye-tracker experiment. The robot was one of the companion robot designs that was developed to investigate the design preferences of the elderly [15]. The bear was designed based on literature regarding companion robot design preference of the older adults [1,15,16,48]. Oh et al. reported that older adults prefer bear as the most among zoomorphic companion robots [16]. Another study also reported that the bear’s toy-like appearance and rounded shape made it seem familiar [15]. Referring to [48], the bear was also the most preferred design of the people in their fifties.

**Figure 1:**
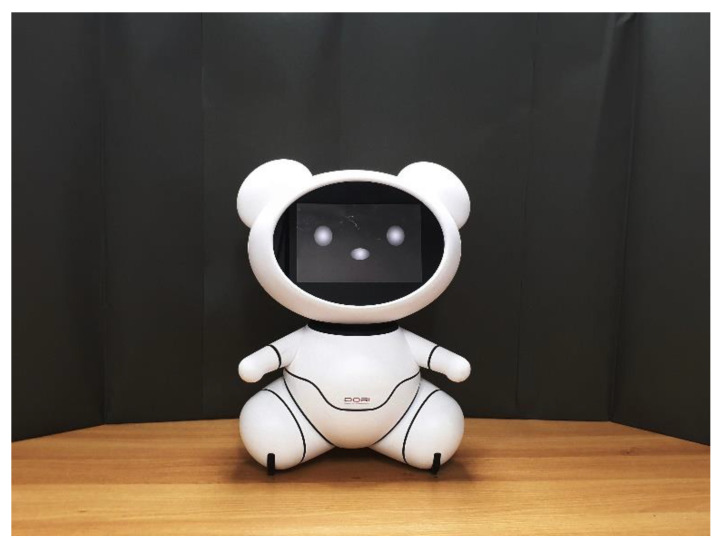
Appearance of the teddy bear-like robot mockup.

The teddy bear-like robot (342 mm × 209 mm × 440 mm, W × D × H) was made of acrylonitrile butadiene styrene material, processed with numeric control machine, and coated with polycarbonate material (Figure 1, see details of its design from [15]).

The bear is composed of several parts (face, arms, legs, wheels, ears, torso). It has a horizontal 20.32 cm (8 in) display on its face. Its facial expression was set to a neutral style [49,50] to control the influence of the robot’s facial expression on the evaluation. The image of the facial expression was printed on a photographic paper and cropped to the display size of the bear. Then, we opened the transparent acrylic display panel of the bear and put the paper behind the panel.

### 3.3. Experimental Setting

**Figure 2:**
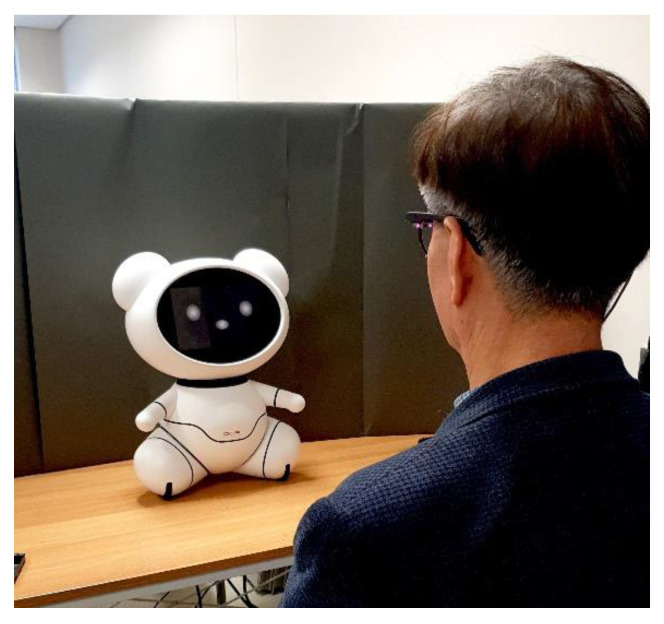
Experimental setting and an older adult looking at the bear-like companion robot.

All experiments were conducted in our laboratory. To avoid the effects of sunlight, windows were covered using blinds during the experiment [47]. The bear was placed in the center of a desk. In order to control the duration of the bear’s exposure, it was covered with a blanket. The distance between the robot and the participant was 80 cm. In previous screen-based eye-tracker studies, the subjects were usually seated at a distance of 50–70 cm from the monitor [26,47,51,52]. However, in this study, when the mockup was 50–70 cm from the participants, it was too large in their field of view. We also considered the presbyopia of older adults [53,54]. Therefore, we increased the distance to 80 cm. In addition, a small wall made by cutting and unfolding a postal box (161 cm × 73 cm) was placed behind the mockup to prevent the participant from being visually distracted (Figure 2). In order to clearly differentiate the bear from the white wall of the laboratory in the participant’s view [55], we placed dark gray Kent paper on the small wall behind the bear (Figure 2).

### 3.4. Procedure

The study was conducted at our laboratory. Participants entered the room and were seated in a chair. They were provided with a brief overview of the experimental procedure and the research purpose. After they signed the informed consent, the study was conducted in the following order:

1. Pre-questionnaire: Prior to responding to the survey, all participants were instructed to read the definition and the role of a companion robot described in the questionnaire [56]. The questionnaire included items about demographic information such as gender, age, and living arrangement. Screening questions were included to check whether the participants had a history of ocular disease.
2. Eye tracking: After the participants completed the questionnaire, they adjusted the chair to a position marked by scotch tape on the floor. We asked them to sit comfortably leaning on the back of the chair. Instructors explained the eye-tracking procedure and how to wear the eye-tracking glasses (Tobii Pro Glasses 2, 50 Hz sampling, resolution: 1920 × 1080 pixels). The instructors asked the participants not to shake their heads as much as possible to facilitate more accurate data collection and analysis [47,57,58]. After confirming that the calibration was successfully completed, one of the instructors removed the blanket from the mockup, and the data collection began. The eye-tracking data were collected for 15 s [8].
3. Post-questionnaire: The participants evaluated the physical attractiveness and the social likeability of the bear on a five-point Likert scale. The questionnaire used was the BEHAVE measurement tool [13], which includes five items of physical attractiveness and five items of social likeability [59]. However, one item—the robot is very sexy looking—was excluded from the physical attractiveness questionnaire because it is beyond the scope of this study. In addition to completing the BEHAVE measurement, they rated the bear’s design on a five-point Likert scale.
4. Individual interview: We asked the participants about the parts of the robot they focused on. They were also interviewed about their opinions about the bear’s design. Next, they were asked to explain the reasons behind their evaluation of the robot’s design. Finally, we asked their opinions about the size of the robot. The interviews lasted about 10 min on average.

### 3.5. Data Analysis

#### 3.5.1. Determining Areas of Interest

**Figure 3:**
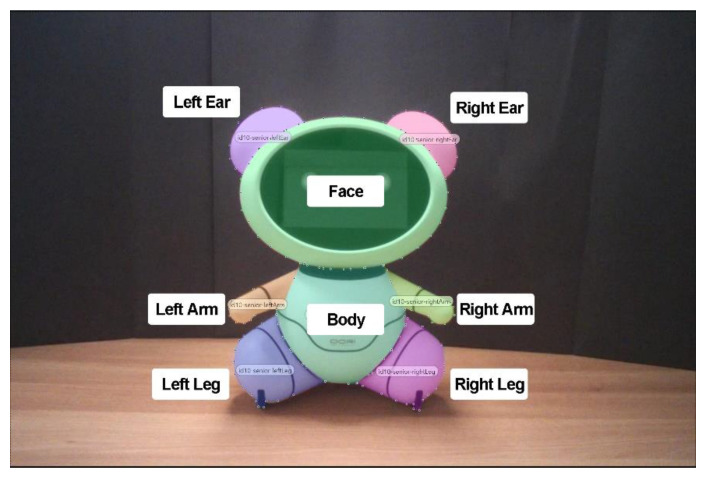
Areas of interest (AOIs) of the bear.

Although age-related differences in eye-fixation pattern on an animal-like robot were partially reported by Choi et al., their preliminary findings were limited by a small sample size (n = 18) [32]. They solely depended on eye-tracking data, thus further interpretation of the results was limited. They did not analyze gaze plots or eye-tracking recordings in detail, thus they could not explain the differences in fixation patterns. Moreover, they presented eye-tracking statistics for the robot’s face, but they did not include other body parts for data analysis. In another study, researchers divided a robot into nine areas of interest (AOIs) and examined which areas people mainly focused on [12]. Based on these findings [12,27,32], the AOIs of the bear were determined as shown in Figure 3. Unlike the robot of [12], the bear’s neck is very short. Thus, it was not included in the analysis.

#### 3.5.2. Eye-Tracking Data Analysis

We analyzed the first 10 s of the eye-tracking data [32,60]. According to [32], in which the authors collected gaze data of participants looking at a zoomorphic robot, the results showed that the gaze data collected for 10 s were very similar to the data recorded for more than 10 s; after the first 10 s, the participants’ eyes remained focused on the robot’s face. Additionally, both the younger and the older adults raised their hands after about 10 s, indicating that they had gazed sufficiently long at the robot to assess its appearance [32].

Previous studies analyzed eye tracking data collected for about 10 s, but there is no consensus in the field on the optimal length of collected gaze data yet. Liu et al. analyzed gaze data collected for 10 s or less for robot images and revealed the preferred humanoid robot design [27]. Al-Samarraie et al. conducted an eye tracker study to investigate the user preferences of graphic design elements [61]. They showed five types of design elements, each for 5 s, to the participants. Furthermore, Park et al. conducted an eye tracker study to investigate facial recognition patterns of robotic faces [33]. In their study, they showed each picture of a robot face for 4 s. Another study showed a short introductory video of an agent for 15 s to investigate participants’ first impression of it [8]. Based on these findings, we analyzed the eye-tracking data recorded for 10 s. We used the Tobii I-VT Fixation filter (Velocity-Threshold Identification Fixation Filter [62]) of Tobii Pro Glasses Analyzer software (version 1.34; Tobii AB, Danderyd, Sweden). The gaze data were mapped onto a still image [63,64] with assisted mapping feature [64,65]. After the assisted mapping was completed, we carefully checked the incorrect mapping points and manually re-mapped them [66,67].

#### 3.5.3. Statistical Analysis

For the statistical analysis, we grouped the sixty-two participants by age as follows: younger adults (aged 18–29, n = 31) and older adults (aged 55–76, n = 31). The independent variable was the age group. The dependent variables were the physical/social attractiveness ratings, the ratings of the bear’s design, and the eye-tracking data: total fixation duration, average fixation duration, and number of fixations. Statistical analyses were completed using SPSS (version 25; IBM Corp, New York, NY, USA) and G*Power 3 (Heinrich-Heine-Universität Düsseldorf, Düsseldorf, Germany). We conducted normality tests for the dependent variables by age groups. Both the Kolmogorov–Smirnov test and the Shapiro–Wilk test showed statistically significant results (*p* < 0.05, data omitted). Therefore, the data did not follow the normal distribution, and we performed Mann–Whitney U tests to compare the two groups. The statistical hypotheses we evaluated were as follows:

- H_1_: The eye-tracking statistics—total fixation duration, average fixation duration, and number of fixations—of each AOI of the robot will differ by age groups.
- H_2_: The rating of the robot’s design will differ by age groups.
- H_3_: The physical attractiveness and the social likeability of the robot will differ by age groups.

## 4. Eye-Tracking and Survey Results

The face and the body attracted most of the overall attention. In particular, the face was the most watched feature regardless of the age group. Moreover, eye-tracking results revealed that the participants’ gaze behavior significantly differed by age group (Table 2), which partially supports H_1_. Older adults looked at the areas around the eyes and the noses very often with little attention paid to the rest of the AOIs. They focused significantly more times and for a longer duration on the face (*p* < 0.01) but significantly less on most of the other AOIs than younger adults. There were no statistically significant differences in some AOIs, but such data were consistent with the overall trend and did not limit the interpretation of the results.

**Figure 4:**
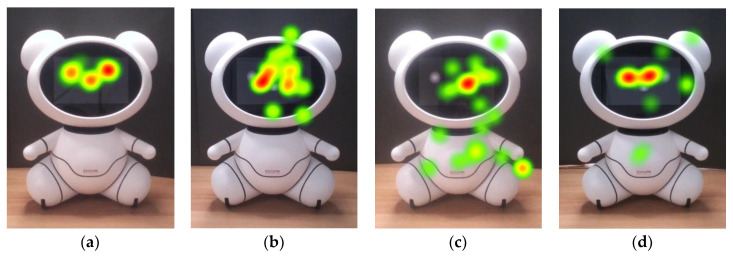
Heatmaps of older adults (**a**) P2, (**b**) P12, (**c**) P17, and (**d**) P22.

Older adults’ high interest in the bear’s face is also supported by the heatmap images in Figure 4. They gazed at the eyes and the noses very often. Indeed, 20 out of 31 older adults never looked at other AOIs but only focused on the face. This is evidenced by the eye-tracking data of the older adults on arms and legs. They focused little attention on the right arm and the left leg (Table 2, Figure 4a,b).

**Figure 5:**
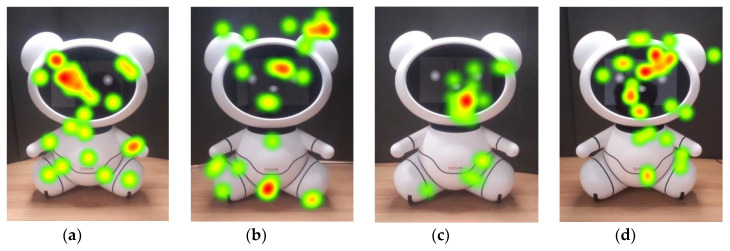
Heatmaps of younger adults (**a**) P2, (**b**) P7, (**c**) P11, and (**d**) P27.

**Figure 6:**
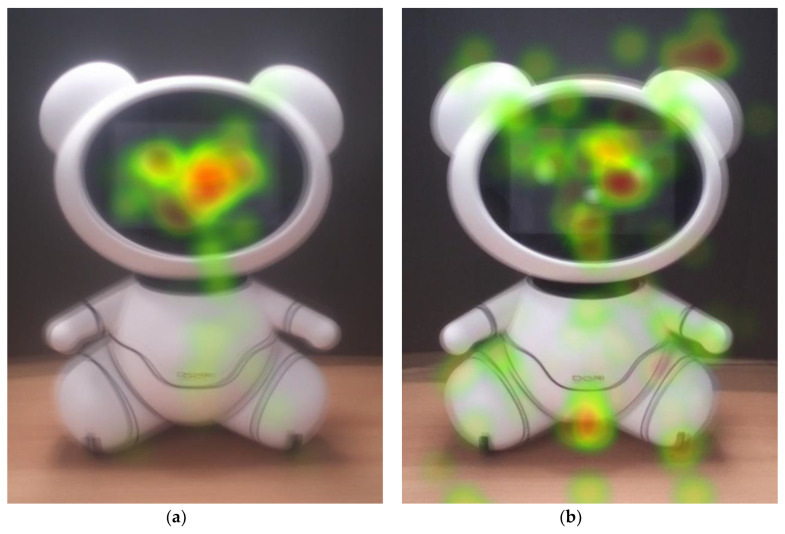
Summary heatmaps of each group: (**a**) older group and (**b**) younger group.

Younger adults also looked at the face for the longest of the entire body, but they looked at the other AOIs more compared to older adults (Figure 5 and Figure 6, and Table 2). Only two of the younger adults focused only on the face (P12 and P14). Table 2 shows that younger adults focused on AOIs other than the face significantly more than the older adults. After the face, the torso drew the most attention. The convex abdomen, the small red colored logo, or the black parting lines may have affected the results, which is further explained in a later section.

**Figure 7:**
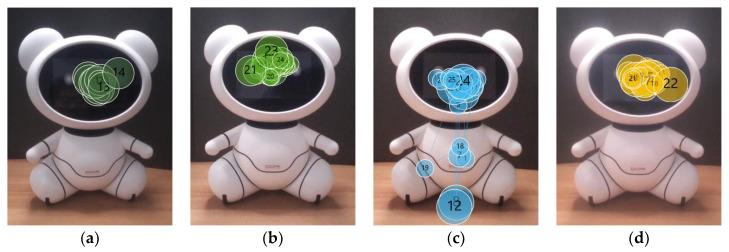
Gaze plots of older adults (**a**) P3, (**b**) P11, (**c**) P19, and (**d**) P30.

The gaze plots also show that the robot’s face drew much attention. Moreover, it was the most popular starting point for the gazing at the robot. Twenty-nine older adults fixated on the face first, while two started by looking at the torso (P17 and P19). The majority of the older participants focused only on the bear’s facial features (n = 20) for the duration of the experiment. Others looked at the other body parts briefly (n = 11). Seven of them viewed the face first and then moved their gaze down to the abdomen; P5 and P13 gazed at the face first and then the ears, while P17 and P19 stared at the bear’s torso first and then at the face (e.g., Figure 7c).

**Figure 8:**
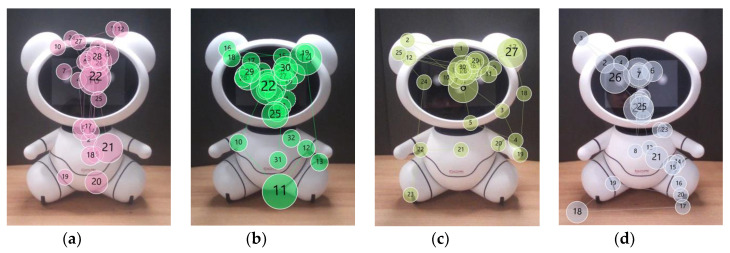
Gaze plots of younger adults (**a**) P6, (**b**) P9, (**c**) P10, and (**d**) P26.

Most younger adults also focused on the face first (n = 22, Figure 8). Then, their focus moved to torso (n = 9), ears (n = 9), or arms (n = 2), or it remained on the face (n = 2). We also observed that, for some younger participants, torso (n = 4), ears (n = 3), arms (n = 1), or legs (n =1) were the starting points of gazing at the robot.

In summary, the eye-tracking data revealed that the bear’s face attracted the most attention, followed by the torso. Older adults showed higher interest in the face than younger adults. They also gazed at fewer of the other AOIs than younger adults.

Survey results showed that the robot was significantly more preferred by older adults (Table 3), which supports H_2_. Its physical attractiveness and social likeability were rated as significantly higher by the older adults (Table 3), which supports H_3_. The rationale behind the preferences for the robot is discussed in a later section.

## 5. Interview Results

We conducted interviews to investigate the participants’ gaze at the robot and examine their impressions in detail. We highlight the age-related differences in their viewpoint of companion-robot design. We report each group’s opinions, focusing on the AOIs that participants primarily gazed at.

### 5.1. Overall Impressions

The impressions of the older adults were quite simple: they loved the bear very much and were fascinated by its design. Moreover, they focused considerably on the bear’s face. The reason they gazed at the face the most was simple as well. Similar to when people look at other people [68,69], the eyes of the older adults were instinctively fixated on the robot’s face:

- *“The first thing I noticed was the bear’s face. Just like when you talk to people, you see them face to face.”*—P25, older.
- *“When you see another person, you first look at their eyes. I think it’s just a habit to have eye contact.”*—P13, older.
- *“Because people see each other’s face and eyes when they first meet, the face naturally comes in the sight first. You can’t just look at their legs first, because that’s not what they’re used to.”*—P11, older.

Then, they recognized the robot as a bear and felt that it looked very similar to a typical teddy bear. Nobody mentioned that the robot’s appearance was unusual.

- *“I recognized the robot as a bear at a first sight. Overall, I think the quality of it is high. It almost looks like a finished product. The balance between the top and bottom parts is good and I personally can’t seem to find a flaw.”*—P5, older.
- *“At the first glance, its shape, a bit like a teddy bear, caught my eye. I know why it caught my eye. Because we are familiar with teddy bears in general, and its overall impression was just like a teddy bear. Eyes, then ears, and then this round face. From these features, I had an instant feeling that this was a teddy bear.”*—P4, older.

Younger adults were also positive toward the robot and stared at its face for quite a long time. However, they tended to look at the robot’s appearance in more detail compared to the older adults. As a result, the older and the younger adults’ viewpoints of the robot differed. Unlike the older adults, the younger adults described many potential improvements of the robot. One example is that approximately one-fifth of the younger adults (n = 6) reported that the robot did not look like a typical bear or teddy bear. In particular, its thin arms and thick legs were one of the causes of that opinion:

- *“I didn’t think it was a bear, maybe because it’s face was too wide? And its body is nothing like what I know of a teddy bear. I think that’s because its arms are thin, and legs are bulky.”*—P14, younger.
- *“On the whole, it is felt a little different from the other bears I’ve been familiar with. The face is too long in its width and the arms and legs also look different. And for legs, you see, one of the main features of a bear is its paws. But there are no such parts with this robot, and I have a kind of wonder whether this is really a bear.”*—P25, younger.

### 5.2. Head

Younger adults criticized that its head was large (n = 5) and wide (n = 4) compared to other body parts, and they felt it looked awkward and unnatural. Some of them added that the face seemed a little bit heavy (n = 3).

- *“When I first saw it, I thought it was cute because its eyes, nose, and mouth reminded me of a teddy bear. But then, later, I thought it was a little weird. (laugh) The face is too big and its facial features are cute yet a little weird.”*—P6, younger.
- *“The face is too.. wide. Haha. I… um, I don’t think it’s good.”*—P9, younger.

On the other hand, most of the older adults viewed its head positively. They were satisfied with the rounded face and ears, which reminded them of teddy bears. There was also an opinion that a large face would be advantageous for older people with low vision to easily see the screen (P24). Only a few of them reported that it was too large (n = 5). Two of them suggested that increasing the size of other body parts (P1) or reducing the size of the head (P20) would lead to a more balanced design: *“I think it’s better to downsize its head to get more balance. I think right now, the upper part is too big.”* — P20, older. Three older participants did not mention the need for modification (P9, P11, P12). P9 felt its face was large, but it still felt cute to her: *“I have a feeling that its face is somewhat large. Having said that, the robot has a cute appearance overall and I only mentioned its large size in case I have to come up with any of its disadvantages. For me, I like the way its eyes appear large from its face, and the large eyes themselves gave me the impression of openness, which was nice.”* — P9, older. In summary, several older adults recognized that the robot’s head was a little large, but they did not consider it a negative aspect of the design.

### 5.3. Body

Older adults believed that the bear had a friendly design. They suggested that the robot’s rounded look, particularly its rounded abdomen, made it seem more friendly.

- *“It is round with no sharp edges, which makes it quite friendly and cute. In general, there are no sharp edges, and its belly, ears, eyes, and face are all round, which makes the bear look friendly and soft. I think the belly poking out is so cute.”*—P23, older.
- *“The belly pokes out, so it looks cute. You know, it reminds me of those mischievous plush toys kids play with. Their bellies are sticking out like this. Or those other toys that look at you like this, showing their belly button. I think it is designed well.”*—P24, older.

The older participants considered the rounded shape (n = 11) and torso (n = 5) of the robot so charming that it formed functional expectations of the robot’s capability in some of them [18], which was addressed only in the older adult’s interviews. Although we did not ask any questions about its perceived role, some of them mentioned that they could talk with the bear (P4, P7, P8) [13]. Others even mentioned that they felt like it could be their friend (P31, P34) [13]. These positive impressions developed into a strong preference for the robot (Table 3).

- *“It’s so cute. It looks like it’s going to talk to me like a little child. Oh, I like that feeling. I think everything about this is cute. I can’t think of anything wrong with it. I feel I can have easy conversations with it like I’m talking to a friend.”*—P7, older.
- *“Overall, it looks cute and funny. I feel it’s going to do something fun with me and talk to me. I feel it’s going to give me a lot of joy.”*—P8, older.
- *“It seems to be bright and affable. I feel it is very friendly just like a puppy, like a pet dog.I feel I should protect this robot and it will be my friend. I think it will run errands for me.”*—P34, older.

The abdomen was also recognized as a friendly feature by younger adults (n = 7). Since it reminded them of the bear dolls or a character (e.g., Winnie-the-Pooh), it reduced the machine-like characteristics of the robot. They were familiar with the abdomen, and that was the reason they focused on it.

- *“Pokey bellies are cute. Winnie-the-Pooh or other plush toys usually have big bellies. I think that’s because people like it. I think that’s why I first looked at its belly. This one feels more friendly, even though it’s a machine, because it has a pokey belly.”*—P5, younger.
- *“Its belly is poking out, so it didn’t occur to me that it would be offensive. In contrast, it felt so friendly, the pokey belly. Like Winnie-the-Pooh. This robot has a similar body shape to littletoddlers.”*—P31, younger.

As shown in Figure 5 and Figure 8, the parting lines and the logo attracted some younger adults’ attention to the torso and other AOIs around it. Perhaps the noticeable colors may have drawn their attention, but those factors had little impact on their impression of the robot. Only a few short comments were made about the parting lines (n = 3) and the logos (n = 2) during the interview. Due to the parting lines, P2 envisioned the modular design and said the robot seemed to be easy to repair. P20 and P23 instinctively stared at the logo to read what is written. Only P30 rated the lines negatively as unnecessary and visually distracting.

### 5.4. Arms and Legs

The arms and the legs did not attract much attention from the older adults. Only a few older participants (n = 5) were interested in them. Their feedback was less specific than that of the younger adults, and most of them were focused on the arms rather than the legs. Although the bear did not show any movement, they viewed the toy-like small arms favorably and felt like holding them:

- *“When I first saw this bear, I thought to myself, I could just hold both of its hands! I felt that I would just hold its hands, if given some kind of assurance that I may hold them.”*—P2, older.

Other older adults mentioned that the arms are a little thin (or short) compared to the legs. They thought it would seem more stable and balanced if the arms were a little thicker (n = 5): *“I think this arm is a bit thin... You see, its feet are as thick as this. If the arms are about twice as thick as they are now, it will look more stable, in better shape overall.”* — P18, older.

Some younger adults’ responses were similar to those of older adults. They also preferred the robot’s arms over its legs (n = 7). To them, it seemed like a doll that wanted to hug them (P31, P33).

- *“The arms were sticking out, just like how other teddy bear plush toys look and beg for your hug. It was cute, so I think that’s why I kept looking at its arms.”*—P33, younger.

However, detailed gaze patterns of younger adults were linked to negative responses on the arms and the legs. More than a third of them pointed out the limbs and criticized the exaggerated size of the limbs (n = 11), stating that it makes the robot seem unnatural and imbalanced. The disproportional limbs also made the robot feel distant from their preconceptions of a bear. Still, a few of them thought it was cute (P17, P24, P29):

- *“The legs looked quite puffy, which makes it unbalanced throughout its arms, belly, and legs. The legs are as large as human. So, I thought it was a little odd, and asked myself whether teddy bears usually look like this. I think the shape is quite.. different from what people generally think of teddy bears. On the positive side, its ugliness makes it cute.”*—P29, younger.
- *“The arms.. do not look like bear’s. They’re too thin.”*—P22, younger.
- *“Right now, it is more like a robot so I think it will be better if it looked a little more natural. This may be difficult because of some parts, but I think it would have looked better with bigger arms.”*—P10, younger.

### 5.5. Size

More than half of the older adults (n = 17) responded that the current size of the bear is suitable for communication. The prevalent opinion among them was that they would be scared if the robot increased in size: *“I feel like the bear is going to follow me wherever I go just like a puppy. With such a feeling, it comes across as a friendly companion. But if its size is too big, I might feel a bit overwhelmed by it. So I think the size is fine as it is. If it is too large, I might be intimidated by it, thinking it could be something… beyond my control.”*— P12, older.

On the other hand, some older adults viewed the current one as too big (n = 7). They thought that the smaller the companion robot is, the more adorable it would be, since it would look like a baby: *“When it’s too big, it’s not cute. The reason why babies are cute is they are small. We all know human babies are so cute, and that’s because they’re so tiny.”* — P11, older. They argued that they would easily talk with the robot, even if it decreased in size. P27 envisioned that the companion robot would be helpful for people living alone. Considering the dimensions of their living space, she guessed that a smaller-sized robot would be suitable for them. P11 and P30 held the same view that the current one would be difficult to place at home due to its size [70].

- *“I think people who live alone would like this bear better. People who live alone usually don’t live in a big house. Since they live in small places, I think it’ll be irrelevant if it’s even smaller than this bear.”*—P27, older.

Some older adults insisted that the size of the robot should be slightly larger than the current size (n = 7). They thought the larger one would be more noticeable at home and it would be more convenient to angle toward the robot (P9, P29). P9 guessed that taller robots would be advantageous when talking with them from a distance.

- *“I think it’ll have to be a little bigger than the current size. I think this is too small. You need to have an even eye level to be able to have conversations. With this size, people will have to bend their back, which makes it uncomfortable. Even if people talk to this bear from a distance, they still need a taller robot.”*—P9, older.
- *“Perhaps a little larger than this one. The robot would look out of place if it were too big. If it is in the house, it needs to be seen easily. In that sense, it should be a little bigger than now.”*—P15, older.
- *“When I stand up, this robot is on the floor, so I can’t see the screen well. Things like the texts on the screen get farther away in those cases, so I think it might be better if its height were a little taller.”*—P18, older.

The opinions of the younger adults were evenly divided into three groups. Ten respondents said the current size of the bear was suitable, ten participants wanted a larger one, and eleven participants preferred a smaller one. Among the three groups, those who criticized its head size demanded the robot be minimized. They hoped to scale its head down and height simultaneously. P24 explained why the bear needed to be smaller - the large head made it look heavy, unstable, and it felt like it would be easily broken if it fell [15]. In agreement with the viewpoint of several older adults, some younger adults thought the small size of the robot would not affect communication, rather it would make the robot seem more friendly.

However, younger adults who preferred a taller robot (n = 10) did not express any opinions about the size of its head. They argued that if the robot navigates at home autonomously, it should be taller to reach eye level. The current size would be so small that they would have to bend at their waist to see the screen, which was also pointed out by the older adults. Some younger adults were concerned that if the robot is too small, it would be regarded as a toy or a simple machine, not a companion (P13, P31). In summary, both groups concluded that the robot needs to reach their eye level.

## 6. Discussion

In this study, we aimed to investigate the age-related differences in attending the design of companion robots. Unlike most of the previous robot design studies that used surveys or interviews [6,15,16,21,23], we used eye-tracking methodology as well. Although surveys and interviews are efficient in collecting data, they may be affected by several types of biases (e.g., response bias) [71]. Beyond self-reported data, recent studies have adopted eye trackers to evaluate product designs [12,24,27,72]. They reported that eye-tracking methodology can objectively investigate the cognitive responses of users [24,26,27,72]. However, the limitation of eye-tracking methodology was also discussed in a previous study [30], i.e., the visually most attractive design is not necessarily the most preferred design. Some researchers suggested that eye-tracking studies should be complemented by a survey [24,30]. Therefore, our study conducted surveys and interviews to complement the eye tracking methodology and understand the impression of the robot design.

It is widely known that, when people look at other people, they look at their faces [68,69,73]; the same is true for humanoid robots [12,27]. Our findings show that the bear’s head drew the most attention, and it was the first area that the participants fixated on. These findings are consistent with [12], which reported that participants gazed at the head the most among the nine AOIs of the humanoid. In addition, the researchers reported that eyes and nose of the humanoid attracted the most of the fixations, which is similar to the gaze behavior people exhibit when looking at other people; they focus on the primary facial features, e.g., eyes and nose [33,68,73]. Such gaze behavior was also observed in our study (e.g., Figure 4 and Figure 7), which supports the claim that this type of gaze behavior is applied to zoomorphic robots.

In this study, younger adults’ detailed gaze behavior seemed to be linked to low preference for the bear. This result is similar to the findings of [15], which reported the negative attitudes of younger adults toward animal-like companion robots. For instance, the younger adults in [15] viewed the bear as unstable and susceptible to breaking in case of a fall. They also disapproved of other animal-like robot designs. Conversely, older adults viewed the bear as stable and familiar [15]. Some of them disapproved of the rabbit-like design concept, but they all approved of the ears of the rabbit, which were criticized as “useless” by the younger adults. In summary, these results indicate that the preferred design and important design features could vary for different age groups.

The bear’s appearance allowed the older participants to attribute positive traits to it. Its rounded shape and abdomen appealed to them. As a result, some older adults came to expect that the robot could perform useful tasks for them (e.g., have a conversation and run an errand) [18], which corresponds with some social likeability questionnaire items from [13]. Some older participants thought this robot might “be a friend of” them [13], and others mentioned that they wanted to have a conversation with it. Moreover, their expectation seems to be linked with “attractiveness halo” [74], which is the psychological tendency of attributing more positive traits to attractive people. As Norman claimed in his book, the rounded shape and the attractive appearance [75] produce positive affects toward the robot, which may be extended to judge the robot as likely to talk and interact with them kindly [19].

Furthermore, baby- or animal-like physical features of the bear might have elicited positive responses from the older adults as argued by Lorenz [76]. Lorenz proposed the concept of a baby schema: a set of cute physical features common to human or animal (e.g., large head, round face, and protruding cheeks) motivates positive social responses, such as nurturance and affection. Hinde and Barden also argued that people respond positively to baby schema features in adults, animals, and human artefacts [77]. In line with these concepts, Breazeal and Forest suggested that cute appearance of a robot could encourage people to react emotionally to the robot [78]. Therefore, the bear’s appearance may have attracted older adults and motivated them to interact with it.

On the other hand, the younger adults did not report such functional expectations of the bear. They were more negative than older adults about the size of its head and exaggerated arms and legs. Their impressions of the bear design were also somewhat different from those of the older adults. In this regard, the bear might have failed to shape such expectations in the younger adults, which supports that there might be age-related differences in recognizing the robot’s appearance.

This study utilized eye-tracking glasses and a mockup for participants to consider the size of the robot when evaluating its physical likeability. This is because design evaluation with two-dimensional images on the monitor is “often too limited” [63] compared to the use of three-dimensional mockup. Further, people usually look at the robot from various points of view. Although the way we utilized wearable eye trackers was different from the typical method of using them—investigating the gaze behavior of participants during movement [24]—our method was effective in eliciting the participants’ opinion on the robot’s size. The majority of the participants thought the current size of the bear was suitable, and they noted that it might be scary if it became larger than its current size. These opinions are similar with the report from [6]. In that study of robot appearance, most of the elderly participants first insisted on the robot’s size from the interviews; they preferred small-sized robots such as Nao (573 mm in height) rather than human-sized robots [6]. Older participants in another study [70] also expressed similar opinions. They expected the robot to be smaller than them and thought that it would fit better in their homes than a larger one. These findings show that the size of the robot is an important element of robot’s design, and older adults tend to prefer small-sized robots [6,22,70,79].

The empirical findings of this study provide several implications for companion robot designers; the preference of prominent physical features and design varies by age. Older adults mostly focused on the companion robot’s face, which is the most important feature for them. In addition, the rounded look and the animal-like or animation character-like appearance may be helpful in forming positive impressions at an early stage. Finally, the size of the robot determines its accessibility. More than half of the older adults favored the current size of the robot, but others hoped to change its height. They also commented that the size of the robot should be determined depending on the robot’s mobility or dimensions of its living space [70].

This study has several limitations. First, the findings are limited to the bear-type robot used in this study. The age-related differences in some AOIs might be attributed to the distinctive design features of this bear-like robot (e.g., large head and convex torso), and the differences may not be consistent if using other animal-like robots. Therefore, further research on different designs and sizes of robots is required.

Second, the results of this study are limited to the participants’ first impressions of the robot design. Their views, investigated through a short eye tracking interaction and brief interview, could be changed by the robot’s interaction capability or long-term usage [80,81]. Therefore, future studies should investigate if interaction time affects participants’ fixation patterns and impressions of the companion robot design (e.g., [8]).

Third, other variables may have influenced the differences in the fixation patterns. In this study, some AOIs received little attention from the older adults, which might be attributed to their cognitive decline. Due to aging, their reaction time and processing speed decrease [40], thus the older adults may have looked at each AOI more slowly than the younger adults [41]. For instance, the average fixation duration of the older group on the bear’s face was slightly longer than that of the younger group (Table 2). The older group also focused on the face significantly more times. Although the average fixation duration on the bear’s face was not significantly different, several previous studies reported that older adults require longer fixation to perceive visual information than younger adults [82,83]. Additionally, they may not have had enough time to look at the other AOIs. If we had collected and analyzed the data for more extended periods of time as in [12], the results might have been different. In this regard, the older adults’ fixation pattern may not be related with their impressions of the robot design. Further research should consider more variables to investigate the relationship between fixation patterns and attitudes toward the robot design.

Fourth, this study was conducted with a small number of participants, and our data may not have enough statistical power to investigate the age-related differences in recognizing the design of companion robot. The effect sizes of most AOIs were medium or large [84,85], but some of them were small (e.g., left arm) or could not be calculated (e.g., right arm and left leg). The effect sizes of some AOIs were small because the participants paid little attention to them. These results indicate that future research should be conducted with longer period of interaction and larger sample sizes.

In addition, future studies should investigate more types of older adults. The older participants in this study were 62.3 years old on average, which is “relatively young” [86], but the impressions of other older adults may vary by age. For instance, older adults aged over 85 and those aged less than 85 had significantly different preferences of companion robot design [16]. Additionally, their impressions might be different depending on their physical or mental health [16].

Moreover, we found several significant differences in the eye-tracking statistics, but in this study, the number of older female adults was more than twice that of the older male adults. Therefore, the data of the older group may have been biased toward the eye-movement behavior of older female participants. When we performed Mann–Whitney U test for older participants by gender, we found no significant differences for AOIs other than the face. The older male adults’ total fixation duration on the face (*M* = 8.050) was significantly longer (*U* = 39, *p* = 0.033) than that of the older female adults (*M* = 6.543), and the older male adults’ average fixation duration on the face (*M* = 0.431) was also significantly longer (*U* = 39, *p* = 0.033) than that of the older female adults (*M* = 0.318). This shows that older male adults focused considerably on the face, but the small number of the older male adults limits the further interpretation of this result. In addition, we performed Mann–Whitney U tests for all participants, but there were no significant differences by gender except for the right arm and the left leg. No significant differences were found between the younger male adults and the younger female adults either. Therefore, further research on the impacts of gender should be conducted in the future.

Furthermore, the older participants’ high preference of the bear might be also attributed to the large proportion of females in the older group (Table 1). Although the Mann–Whitney U test found no significance for the bear design preference by gender (*U* = 55, *p* = 0.147), the bear tended to be more preferred by the older female adults (*M* = 4.583) than the older male adults (*M* = 4.286). The survey results of the BEHAVE questionnaire were also consistent with the results of the design preference. The Mann–Whitney U test showed that older female adults (*M* = 21.875) evaluated the social attractiveness of the robot as higher (*U* = 33.5, *p* = 0.016) than older male adults (*M* = 19.286). We found no significant difference for physical attractiveness (*U* = 74.5, *p* = 0.647), but it was evaluated as higher by older female adults (*M* = 17.083) than by older male adults (*M* = 16.571). Therefore, with a larger sample size and different statistical analyses, the effects of gender and other factors could be investigated in future work.

Finally, the quality of the collected gaze data might have been influenced by eye tracker slippage. Due to the nature of the head-worn eye tracker, the movement of the facial muscles might have affected the data quality [46].

## 7. Conclusions

Previous studies conducted surveys or interviews to investigate the design preferences of companion robots. In our study, we conducted an eye-tracking experiment on a bear-like robot mockup to objectively investigate the impressions of the robot design. We also conducted surveys and interviews to investigate the viewpoints of older adults. This study aimed to investigate the age-related differences in gaze behavior and design impressions of a bear-like companion robot. Using eye-tracking technology, we found several age-related differences in gazing at the robot. Older adults consistently looked at its face, whereas younger adults gazed at the other AOIs in addition to its face. In the survey and the interview, both groups reported favorable opinions toward the robot, but the younger adults showed more negative attitudes toward the design of the robot. They also provided more detailed feedback on each AOI than the older adults. Both the survey and the interview showed that the robot was significantly more preferred by the older adults than the younger adults. Some older adults formed functional expectations about the robot based on its attractive appearance. In conclusion, our findings suggest that companion-robot designers need to consider the age-related differences in recognizing robot design. However, the findings of this study are limited to the bear-type robot and participants’ first impressions of it. Further research on different designs of companion robots may be required. With larger sample size and different statistical methods, the effects of other variables could be also investigated in future work.

## Figures and Tables

**Table 1:** Demographic information of the participants.

Demographic Information	Age Group	
	Older	Younger
number of participants	31	31
number of male participants	7	16
number of female participants	24	15
mean age in years (range)	62.3 (55–76)	23.3 (18–29)

**Table 2:** Eye-tracking statistics comparison by AOIs: the Mann–Whitney U test.

AOI	Dependent Variable	Group	Mean	U	Sig.	Effect Size (d)
Face	Total fixation duration (s)	younger	4.271	163.00	0.000 ^1^	1.357
		older	6.883			
	Average fixation duration (s)	younger	0.302	411.00	0.327	0.354
		older	0.344			
	Number of fixations	younger	14.194	180.00	0.000 ^1^	1.257
		older	21.065			
Left ear	Total fixation duration (s)	younger	0.088	341.00	0.003 ^1^	0.667
		older	0.008			
	Average fixation duration (s)	younger	0.084	342.00	0.003 ^1^	0.642
		older	0.008			
	Number of fixations	younger	0.355	340.50	0.003 ^1^	0.787
		older	0.032			
Right ear	Total fixation duration (s)	younger	0.139	373.00	0.039 ^1^	0.619
		older	0.023			
	Average fixation duration (s)	younger	0.100	376.00	0.044 ^1^	0.604
		older	0.023			
	Number of fixations	younger	0.452	381.50	0.056	0.543
		older	0.129			
Body	Total fixation duration (s)	younger	1.144	234.50	0.000 ^1^	0.747
		older	0.383			
	Average fixation duration (s)	younger	0.254	205.00	0.000 ^1^	1.085
		older	0.076			
	Number of fixations	younger	3.613	267.00	0.002 ^1^	0.600
		older	1.774			
Left arm	Total fixation duration (s)	younger	0.083	402.50	0.046 ^1^	0.451
		older	0.011			
	Average fixation duration (s)	younger	0.075	402.00	0.044 ^1^	0.472
		older	0.006			
	Number of fixations	younger	0.226	405.50	0.054	0.371
		older	0.065			
Right arm	Total fixation duration (s)	younger	0.090	372.00	0.005 ^1^	Infinite ^2^
		older	0.000			
	Average fixation duration (s)	younger	0.065	372.00	0.005 ^1^	Infinite ^2^
		older	0.000			
	Number of fixations	younger	0.323	372.00	0.005 ^1^	Infinite ^2^
		older	0.000			
Left leg	Total fixation duration (s)	younger	0.190	232.50	0.000 ^1^	Infinite ^2^
		older	0.000			
	Average fixation duration (s)	younger	0.124	232.50	0.000 ^1^	Infinite ^2^
		older	0.000			
	Number of fixations	younger	0.774	232.50	0.000 ^1^	Infinite ^2^
		older	0.000			
Right leg	Total fixation duration (s)	younger	0.144	288.00	0.000 ^1^	0.980
		older	0.004			
	Average fixation duration (s)	younger	0.090	288.00	0.000 ^1^	1.027
		older	0.004			
	Number of fixations	younger	0.710	291.50	0.000 ^1^	0.937
		older	0.032			

^1^ Shows a significant difference, ^2^ Effect size could not be calculated because the standard deviation of the group was zero.

**Table 3:** Results of questionnaire by age group: Mann–Whitney U Test.

Dependent Variable	Group	Mean	U	Sig.	Effect size (d)
Design Preference	younger	3.665	155.0	0.000 ^1^	1.515
	older	4.516			
BEHAVE physical attractiveness	younger	13.839	198.0	0.000 ^1^	1.216
	older	16.968			
BEHAVE social likeability	younger	18.419	221.0	0.000 ^1^	0.971
	older	21.290			

^1^ Shows a significant difference.
